# Transgenerational impacts of herbivory and inbreeding on reproductive output in *Solanum carolinense*

**DOI:** 10.1002/ajb2.1402

**Published:** 2020-01-15

**Authors:** Chad T. Nihranz, William S. Walker, Steven J. Brown, Mark C. Mescher, Consuelo M. De Moraes, Andrew G. Stephenson

**Affiliations:** ^1^ Intercollege Graduate Program in Ecology Pennsylvania State University University Park PA 16802 USA; ^2^ Department of Biology Pennsylvania State University University Park PA 16802 USA; ^3^ Department of Environmental Systems Science Swiss Federal Institute of Technology (ETH Zurich) CH‐8092 Zurich Switzerland

**Keywords:** fitness, flower and fruit production, herbivory, horsenettle, inbreeding depression, *Manduca sexta*, maternal effects, seed germination, Solanaceae, transgenerational effects

## Abstract

**Premise:**

Plant maternal effects on offspring phenotypes are well documented. However, little is known about how herbivory on maternal plants affects offspring fitness. Furthermore, while inbreeding is known to reduce plant reproductive output, previous studies have not explored whether and how such effects may extend across generations. Here, we addressed the transgenerational consequences of herbivory and maternal plant inbreeding on the reproduction of S*olanum carolinense* offspring.

**Methods:**

*Manduca sexta* caterpillars were used to inflict weekly damage on inbred and outbred *S. carolinense* maternal plants. Cross‐pollinations were performed by hand to produce seed from herbivore‐damaged outbred plants, herbivore‐damaged inbred plants, undamaged outbred plants, and undamaged inbred plants. The resulting seeds were grown in the greenhouse to assess emergence rate and flower production in the absence of herbivores. We also grew offspring in the field to examine reproductive output under natural conditions.

**Results:**

We found transgenerational effects of herbivory and maternal plant inbreeding on seedling emergence and reproductive output. Offspring of herbivore‐damaged plants had greater emergence, flowered earlier, and produced more flowers and seeds than offspring of undamaged plants. Offspring of outbred maternal plants also had greater seedling emergence and reproductive output than offspring of inbred maternal plants, even though all offspring were outbred. Moreover, the effects of maternal plant inbreeding were more severe when plant offspring were grown in field conditions.

**Conclusions:**

This study demonstrates that both herbivory and inbreeding have fitness consequences that extend across generations even in outbred progeny.

Plants provision their seeds with material resources such as proteins, nutrients, and energy storage products, as well as with information transmitted via hormones and RNA transcripts (Westoby and Rice, 1982; Bazzaz et al., 1987; Frey et al., 2004). The quality and quantity of these provisions can have major impacts on offspring success, and maternal effects have been shown to influence seed dormancy, germination success, seedling establishment, and the competitive ability of juvenile plants (e.g., Roach and Wulff, 1987; Donohue, 2009). Biotic and abiotic stresses can interfere with seed provisioning and thereby reduce seed quality. Herbivory, for example, can decrease photosynthetically active leaf area and deplete plant nutrient reserves (Nabity et al., 2009), thus reducing the availability of resources maternal plants can provide to their developing seeds. Furthermore, there is evidence that herbivory on maternal plants can influence offspring defense phenotypes, herbivore resistance, and life history traits (Agrawal, 2001; Holeski, 2007; Steets and Ashman, 2010; Rasmann et al., 2012; Colicchio, 2017). These transgenerational responses to herbivory may be mediated through maternal resource provisioning to the seed, small molecule signaling, or by environmentally induced epigenetic changes to the plant genome (see reviews by Roach and Wulff, 1987; Herman and Sultan, 2011). Yet, while the impacts of herbivory on plant fitness have been well studied (e.g., Crawley, 1989, 1992, 1997; Louda, 1989; Gange, 1990; Marquis, 1992; Strauss and Zangrel, 2002), little work has explored how herbivory on maternal plants may influence the growth and reproduction of offspring (but see Agrawal, 2001, 2002; Steets and Ashman, 2010; Holeski et al., 2013; González‐Megías, 2016).

Insect herbivory can have severe negative effects on plant growth, development, and reproduction (Crawley, 1989, 1992, 1997; Louda, 1989; Gange, 1990; Marquis, 1992; Strauss and Zangrel, 2002). Some of these fitness‐related costs are due to direct predation of plant reproductive structures (Janzen, 1971; Crawley, 1992; Louda and Potvin, 1995; McCall and Irwin, 2006; González‐Megías, 2016), while others are associated with trade‐offs that result in the reallocation of resources away from plant growth and reproduction and toward either compensation for lost leaf material or the induction of anti‐herbivore defenses (Herms, 1992; Trumble et al., 1993; Cipollini et al., 2003; Huot et al., 2014; Züst and Agrawal, 2017; Nihranz et al., 2019). For example, chewing insect herbivores can induce plant defenses that directly interfere with herbivore feeding (e.g., spines, trichomes, secondary metabolites) and indirect defenses that attract natural enemies of the herbivores (e.g., volatile organic compounds) (De Moraes et al., 1998; Paré and Tumlinson, 1999; Kessler and Baldwin, 2002; Holeski, 2007; Kariyat et al., 2013; Barton, 2015). Induced defenses allow plants to direct resources toward growth and reproduction when conditions are favorable (i.e., absent/low herbivore pressure), while reallocating resources toward anti‐herbivore defenses during herbivore attack (Karban and Myers, 1989; Agrawal, 1998; Schuman et al., 2012; McArt et al., 2013). Recent studies have shown that inbreeding can compromise plant responses to herbivory, including the induction of defenses against herbivores (see review by Carr and Eubanks, 2014). However, no previous studies have explored how inbreeding influences transgenerational effects of herbivory on plant phenotypes.

Inbreeding is common in flowering plants (Barrett and Eckert, 1990) and has the potential to affect the quantity and quality of resources that a maternal plant can provide to offspring (Husband and Schemske, 1996; Hayes et al., 2005). Self‐fertilization decreases heterozygosity, thereby exposing deleterious recessive alleles to selection and simultaneously decreasing the contribution of overdominance to fitness (Charlesworth and Charlesworth, 1987). Consequently, inbred progeny often exhibit inbreeding depression such as reduced germination and growth, slower development, and decreased reproductive output relative to outbred plants (e.g., Husband and Schemske, 1996; Baskin and Baskin, 2015). Inbred plants also typically show reduced expression of constitutive and induced defense‐related traits (Kariyat et al., 2012, 2013; Leimu et al., 2012; Campbell et al., 2014), which can result in reduced resistance to herbivores (Carr and Eubanks, 2002; Ivey et al., 2003; Leimu et al., 2008; Delphia et al., 2009; Bello‐Bedoy and Núñez‐Farfán, 2010; Kariyat et al., 2011). Furthermore, the magnitude of inbreeding depression is environmentally dependent, and its consequences have been shown to be more severe when plants are exposed to environmental stress, including herbivory (e.g., Armbruster and Reed, 2005; Hayes et al., 2005).

Aside from maternal plant resource provisioning, epigenetic modifications have also been shown to play a role in the transgenerational response of plants to herbivory (Jablonka and Raz, 2009; Herman and Sultan, 2011; Holeski et al., 2012). Epigenetic modifications can alter gene expression through DNA methylation, histone modifications, and small‐RNA biosynthesis, and are known to regulate plant growth, development, and reproduction (Pikaard and Scheid, 2014; Campos‐Rivero et al., 2017). Previous studies have shown that both biotic and abiotic stresses can induce heritable epigenetic modifications in plants that can affect offspring phenotypes (see reviews by Holeski et al., 2012; Thiebaut et al., 2019). Furthermore, there is evidence that epigenetic modifications play a role in inbreeding depression (Vergeer et al., 2012).

Research on the transgenerational impacts of herbivory have primarily focused on the expression of plant defensive traits in offspring. Relatively few studies have addressed the transgenerational effects of insect herbivory on offspring fitness, and no studies have explored how inbreeding influences these transgenerational effects. This study examines the impact of *Manduca sexta* herbivory and maternal plant inbreeding on the growth and reproduction of *Solanum carolinense* offspring. Our specific goals were to determine (1) whether herbivory on maternal plants of *S. carolinense* affects seedling emergence, flower production, and reproductive output of the offspring and (2) whether maternal plant inbreeding influences the transgenerational effects of herbivory on fitness components of outbred offspring. To address these questions, we used greenhouse and field experiments to assess seedling emergence, flower production, fruit production, and seed set of outbred *S. carolinense* offspring from herbivore‐damaged and undamaged, inbred and outbred maternal plants.

## MATERIALS AND METHODS

### Study system

*Solanum carolinense* L. (Solanaceae) is an herbaceous, perennial weed common throughout the eastern United States and southeastern Canada (Britton and Brown, 1970). It is a pioneer species of early successional habitats, waste places, crop fields, and pastures. Once established, *S. carolinense* spreads via horizontal rhizomes that can extend a meter or more from the parent stem (Ilnicki et al., 1962). These rhizomes overwinter belowground and produce new shoots in the spring. Flowering begins during the summer and continues until the first hard frost. The inflorescences of *S. carolinense* are open cymose racemes consisting of 5–20 flowers. The flowers are buzz‐pollinated by bumblebees and carpenter bees, which vibrate the anthers to remove pollen (Hardin et al., 1972). The fruit is a smooth and glabrous berry that is yellow‐orange at maturity and typically contains 60–100 seeds (Bassett and Munro, 1986). *Solanum carolinense* is considered an economically and agriculturally important weed because it acts as an alternate host for insect herbivores and diseases of closely related crops in the genus *Solanum* (e.g., tomato, eggplant, and potato) (Ilnicki et al., 1962; Bassett and Munro, 1986). *Solanum carolinense* is attacked by a variety of specialist herbivores (e.g., *Epitrix fuscula* [Chrysomelidae], *Leptinotarsa junta* [Chrysomelidae], and *Manduca sexta* [Sphingidae]) (Imura, 2003; Wise, 2007) and has a variety of physical and chemical traits that likely play a role in defense against herbivores. The leaves and stems are covered with spines, leaves are also covered with stellate trichomes, and all plant parts including leaves, flowers, and fruits contain constitutive, as well as inducible, toxic secondary compounds (e.g., glycoalkaloids) (Bassett and Munro, 1986; Cipollini and Levey, 1997; Cipollini et al., 2002).

*Solanum carolinense* has a solanaceous‐type, RNAse‐mediated gametophytic self‐incompatibility system controlled by the multiallelic *S*‐locus (Richman et al., 1995). However, there is plasticity in this self‐incompatibility system. The ability of *S. carolinense* to produce selfed seeds increases with the age of unpollinated flowers and when fruit production is low (Stephenson et al., 2003; Travers et al., 2004). Additionally, plants possessing certain *S*‐alleles have higher levels of self‐compatibility (Mena‐Alí and Stephenson, 2007). Because of this plasticity in self‐incompatibility, *S. carolinense* does self‐fertilize under field conditions and experiences inbreeding depression in the greenhouse and field (Mena‐Ali et al., 2008; Kariyat et al., 2011).

### Insects

The tobacco hornworm (*Manduca sexta* L.) is a specialist lepidopteran herbivore of solanaceous plants and is a common herbivore of *S. carolinense* throughout its range (Imura, 2003; Wise, 2007; Delphia et al., 2009). *Manduca sexta* eggs (Carolina Biological, Burlington, NC, USA) were hatched in translucent 32 oz. plastic containers, and larvae were reared on an artificial wheat germ‐based diet (Frontier Agricultural Sciences, Newark, DE, USA) for multiple generations. Each summer, wild *M. sexta* larvae were collected from tomato fields at the Russell E. Larson Agricultural Research Farm in Rock Springs, Pennsylvania. Wild larvae were reared in the laboratory to adults and mated with adult *M. sexta* from the lab colony to increase genetic diversity of the lab colony. All caterpillars used in this experiment were newly molted 4^th^ instar *M. sexta* larvae.

### Plant material

Plants used in this experiment were collected from a large *S. carolinense* population occupying an approximate 180 ha area near State College, Pennsylvania (40°48′28.4″N, 77°52′29.6″W). To reduce the possibility of collecting cuttings from the same genet, we took rhizome cuttings from 16 plants that were at least 10 m apart. These cuttings were resprouted in 4‐L pots in a greenhouse and allowed to grow and flower. After flowering, shoots were cut back, and the pots were put in a 4°C cold room for 6–8 wk. Ramets were generated from rhizome cuttings from the 16 plants, replanted in 4‐L pots, and allowed to grow in a greenhouse. Flowers from each ramet were hand‐pollinated to produce self and cross seeds. The resulting seeds were germinated and grown in a greenhouse. Plants were cut back, and the rhizomes were stored in a cold room at 4°C (for details see Mena‐Alí, 2006; Mena‐Alí and Stephenson, 2007). Various subsets of the resulting selfed and crossed plants were used in a series of studies by our group.

### Experimental design

Three (of the original 16) maternal families were selected for this study (A5, A7, B9). None of the three families selected had an *S*‐allele in common, indicating that they were not clonal replicates and that they are unlikely to be close relatives (Mena‐Alí and Stephenson, 2007). Within each family, three self‐pollinated and three cross‐pollinated genets (i.e., genotypes) were selected for a total of 18 individual genets. Two ramets were created from each genet by taking 2.5 cm cuttings from the rhizomes and resprouting them in flats of potting soil (Pro‐Mix, Premier Horticulture, Quakertown, PA, USA) in a growth chamber (16 h light/8 h dark, 25°C/22°C, 65% RH). After 3 wk, the resprouts were individually transplanted in 4‐L pots. Ramets continued to grow for 4 wk and then were randomly assigned to either a control treatment (no damage) or herbivore‐damage treatment (damaged by *M. sexta* larvae).

All plants in the herbivore‐damage treatment were subjected to 18 bouts (2 bouts per wk for 9 wk) of *M. sexta* feeding damage. Plants assigned to the undamaged, control group did not receive any type of damage. Before each bout of damage, early 4^th^ instar larvae were starved for 4 h. During each damage application, two randomly selected larvae were placed on lower leaves of each *S. carolinense* plant assigned to the herbivore‐damage treatment group and were allowed to feed ad libitum for 4 h. Damage applications started before flowering and continued until all plants had produced mature fruit. No caterpillar was used on more than one plant.

To produce seeds from herbivore‐damaged and undamaged parent plants, hand‐pollinations were performed on 20 flowers from each plant. Plants in the undamaged control group were cross‐pollinated with pollen from other undamaged plants, and plants in the herbivore‐damage treatment group were cross‐pollinated with pollen from other herbivore‐damaged plants. These cross‐pollinations ensured that all next‐generation offspring grown from seed were outbred and had a coefficient of inbreeding equal to 0 (ƒ = 0). Thus, any fitness‐related effects of inbreeding observed in the offspring are the result of the maternal plant breeding type.

Twelve weeks after the first damage application, all plants had produced mature fruit and were harvested. Fruit were harvested from each plant, and seeds were extracted. Seeds were weighed, and the total number of seeds produced by each plant was estimated by dividing total seed mass per plant by the mass of 25 randomly selected mature seeds from that plant.

### Seedling emergence and flower production in the greenhouse

To determine the effect of previous generation herbivory and maternal plant inbreeding on components of *S. carolinense* offspring fitness in the absence of herbivores, we assessed seedling emergence and flower production in the greenhouse. Eight to 24 seeds from each genotype were planted in flats of potting soil (Pro‐Mix, Premier Horticulture) in a pest‐free greenhouse (16 h light/8 h dark, 25°C/22°C, 65% RH). To assess seedling emergence, we counted the emerged seedlings daily for 30 d. This seedling emergence experiment was replicated four times (July 2015, November 2015, February 2016, and March 2016) for a total of 40–52 seeds per genotype (1756 seeds total). In all replicates, the seeds of one undamaged inbred ramet never germinated, so that plant and the damaged inbred counterpart were removed from analyses.

Flower production was examined in the greenhouse on seedlings that were planted in July 2015. Thirty days after planting, seedlings were transplanted into 2‐L pots with potting soil, given 6 g Osmocote Plus fertilizer (15‐9‐12 NPK, plus micronutrients, Scotts Co., Marysville, OH, USA) and allowed to grow. To determine the effect of previous generation herbivory and maternal plant inbreeding on flower production and timing, we counted the number of flowers produced per plant for 15 d starting at the onset of flowering. These plants were then used for another experiment examining the transgenerational effects of herbivory and maternal plant inbreeding on defense‐related traits in *S. carolinense* offspring (not reported here).

### Flower production, fruit production, and seed set in the field

To determine the effect of previous generation herbivory and maternal plant inbreeding on offspring reproductive traits under natural conditions, we assessed flower production, fruit production, and seed set in the field. Before the start of the field experiment, seeds were planted in flats of potting soil (Pro‐Mix, Premier Horticulture) in a pest‐free greenhouse (16 h light/8 h dark, 25°C/22°C, 65% RH). After 30 d, emerged seedlings were transplanted into 2‐L pots and given 3 g Osmocote Plus fertilizer. Plants were grown in the greenhouse for an additional 8 wk before they were transplanted into two fields at the Russell E. Larson Agricultural Research Farm in Rock Springs, Pennsylvania (40°42′36.7″N, 77°57′52.2″W). In total, 58 (45%) herbivore‐damaged outbred plants, 32 (25%) undamaged outbred plants, 27 (21%) herbivore‐damaged inbred plants, and 13 (10%) undamaged inbred plants were transplanted into the field, which is a fair representation of the general greenhouse population structure based on the proportion of emerged seedlings (herbivore‐damaged outbred = 33%, undamaged outbred = 29%, herbivore‐damaged inbred = 22%, undamaged inbred = 16%). Of these, 39%, 32%, and 29% of field plants were from the A5, A7, and B9 maternal plant families, respectively. These proportions were also a fair representation of the general greenhouse population structure (A5 = 32%, A7 = 39%, B9 = 29%). Offspring planted into the field represented individuals from each treatment within each maternal family and had not yet produced flowers or flower buds.

The new flowers produced by each plant were counted every 3–4 d starting at the onset of flowering and continued for 13 wk. After the first frost in October, the total number of mature fruit produced per plant was recorded. All mature fruit were collected, and their seeds were extracted. Seeds were weighed, and the total number of seeds produced per plant was estimated by dividing the total seed mass by the mass of a random sample of 25 mature seeds. The average number of seeds produced per fruit was then calculated by dividing the estimated total number of seeds produced per plant by the total number of mature fruit on that plant.

### Statistical analyses

All analyses were performed with R statistical software (R Core Team, 2013). A log‐likelihood ratio test of independence was performed to test the effects of parental herbivore‐damage treatment (herbivore‐damaged vs. undamaged), maternal breeding type (outbred vs. inbred), and maternal plant family on the proportion of seedlings that emerged in the greenhouse and the proportion of plants that produced flowers and fruit in the field. Linear mixed‐effects model ANOVAs (lmer function) were used to examine the effects of herbivore‐damage treatment, maternal breeding type, and maternal plant family on the total number of seeds and individual seed mass produced by maternal plants, offspring flower production in the greenhouse, and offspring flower, fruit, and seed production in the field (Bates et al., 2015). All models included the main effects of parental herbivore‐damage treatment (fixed), maternal breeding type (fixed), the breeding by damage interaction term (fixed), and maternal plant family (random). In all experiments done in the field, field plot was used as a random blocking factor in the models. Data transformations were performed when needed to meet the assumptions of each statistical test. Analyses for average number of flowers and average number of fruit included all plants and data were log+1 transformed. To determine whether the random effect of maternal plant family was significant in its associated models, performances for models with and without the random family effect were compared using likelihood ratio tests for all linear mixed‐effects model ANOVAs. Post hoc comparisons using least square means multiple comparisons (lsmeans function) were performed to examine differences among means for all fixed interactions terms with *P*‐values adjusted for multiple comparisons (Lenth, 2016). Linear mixed‐effects model ANOVAs were also run separately for offspring of outbred and inbred maternal plants to assess the effects of previous generation herbivory within each maternal breeding type. All figures were created with the ggplot2 package in R (Wickham, 2016).

## RESULTS

### Maternal plant seed production is negatively affected by inbreeding, but not by herbivory

In the growth chamber, outbred plants produced significantly more seeds than inbred plants (outbred = 1296 ± 104 seeds, inbred = 621 ± 104 seeds, LSMeans ± SE; Table 1). However, there was no effect of herbivore damage (herbivore‐damaged = 950 ± 104 seeds, undamaged = 968 ± 104 seeds, LSMeans ± SE; Table 1) or maternal plant family [*χ* ^2^(1) < 0.001, *P* = 1] on the total number of seeds produced by maternal plants. Seed mass was not affected by herbivore damage, maternal breeding type, their interaction, or maternal plant family [Table 1; Family: *χ* ^2^(1) = 0.04, *P* = 0.845].

**Table 1:** Linear mixed‐effects ANOVA for the effect of herbivore‐damage treatment, maternal breeding type, and their interaction on the total number of seeds produced per maternal plant and the average mass of individual seeds. *P* values < 0.05 are in boldface.

Plant trait	Source of variation	df	SS	*F*	*P*
Number of seeds	Damage	1	2862	0.015	0.904
	Breeding	1	4113460	21.168	**<0.001**
	Breeding × Damage	1	10235	0.053	0.820
	Error	30	5880847		
Seed mass (mg)	Damage	1	0.001	0.014	0.907
	Breeding	1	0.134	1.665	0.207
	Breeding × Damage	1	0.028	0.344	0.562
	Error	30	2.422		

### Seeds from herbivore‐damaged plants and outbred maternal plants have higher rates of seedling emergence

**Figure 1:**
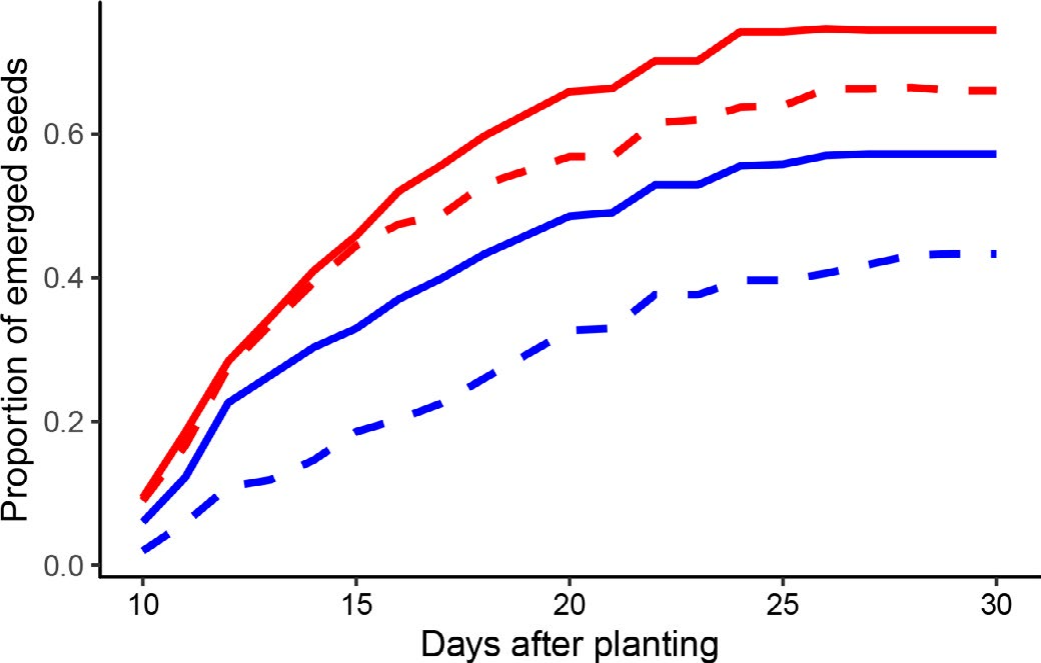
Proportion of seedlings that emerged from seeds of herbivore‐damaged outbred (solid, red), undamaged outbred (dashed, red), herbivore‐damaged inbred (solid, blue), and undamaged inbred (dashed, blue) *Solanum carolinense* plants grown in the greenhouse.

**Figure 2:**
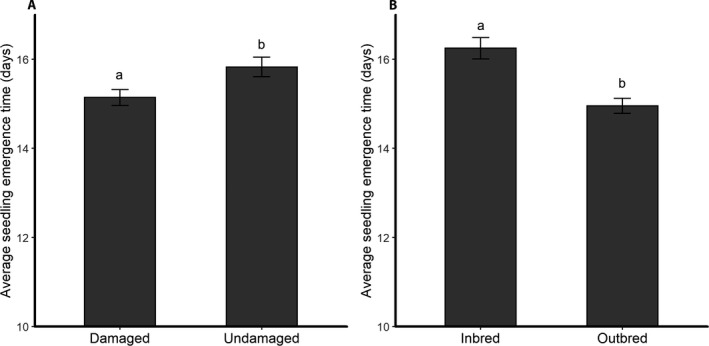
Average seedling emergence time (days) of offspring from (A) herbivore‐damaged and undamaged and (B) inbred and outbred *Solanum carolinense* parent plants. Different letters indicate significant differences between (A) herbivore‐damage treatments and (B) maternal breeding types determined by a linear mixed‐effects model ANOVA. Error bars correspond to standard errors.

*Manduca sexta* herbivory had a significant effect on seedling emergence, with seedlings from herbivore‐damaged plants emerging at a significantly higher proportion (Fig. 1, Table 2) and earlier (Fig. 2A, Table 3) than seedlings from undamaged parent plants. Seeds from outbred maternal plants also had a significantly higher total emergence (Fig. 1, Table 2) and emerged significantly earlier (Fig. 2B, Table 3) than seeds from inbred maternal plants.

**Table 2:** Log‐likelihood ratio test of independence for the effect of herbivore‐damage treatment, maternal breeding type, their interaction, and maternal plant family on the proportion of *Solanum carolinense* seedlings that emerged. *P* values < 0.05 are in boldface.

Source of variation	df	*G* ^2^	*P*
Damage	1	21.964	**<0.001**
Breeding	1	69.237	**<0.001**
Breeding × Damage	3	97.404	**<0.001**
Family	2	12.131	**0.002**

**Table 3:** Linear mixed‐effects ANOVA for the effect of herbivore‐damage treatment, maternal breeding type, and their interaction on the seedling emergence time (days). *P* values < 0.05 are in boldface.

Plant trait	Source of variation	df	SS	*F*	*P*
Seedling emergence time	Damage	1	471.85	34.688	**<0.001**
	Breeding	1	522.19	38.389	**<0.001**
	Breeding × Damage	1	210.43	15.470	**<0.001**
	Error	1069	14541.13		

There was a significant breeding by damage interaction effect on seedling emergence (Fig. 1, Table 2), which was due to an underperformance of the seedlings from undamaged inbred plants. When comparing seedling emergence within each breeding type, seeds from herbivore‐damaged parent plants had a significantly higher proportion of emergence than seeds from undamaged parent plants for both outbred (herbivore‐damaged outbred = 74%, undamaged outbred = 66%; *G* ^2^ = 8.21, df = 1, *P* = 0.004) and inbred maternal plants (herbivore‐damaged inbred = 57%, undamaged inbred = 43%; *G* ^2^ = 15.88, df = 1, *P* < 0.001). There was a significant breeding by damage interaction effect on the timing of seedling emergence (Table 3), which was also due to an underperformance of the seedlings from undamaged inbred plants. Seedlings from undamaged inbred plants emerged significantly later than seedlings from herbivore‐damaged inbred plants and seedlings from herbivore‐damaged and undamaged outbred plants (herbivore‐damaged outbred = 16.2 ± 1.3 days, undamaged outbred = 16.7 ± 1.3 days, herbivore‐damaged inbred = 16.8 ± 1.3 days, undamaged inbred = 19.1 ± 1.3 days, LSMeans ± SE). Finally, maternal plant family had a significant effect on the total proportion of emerged seedlings (Table 2), but not on seedling emergence time [*χ* ^2^(1) = 1.92, *P* = 0.165].

### Offspring of herbivore‐damaged plants produce more flowers and flower earlier under greenhouse conditions

Herbivory in the previous generation significantly affected the proportion of offspring that flowered (Table 4) and average time to first flower (Table 5) in the greenhouse. Offspring of herbivore‐damaged parents flowered at a higher proportion (herbivore‐damaged = 54.4%, undamaged = 42.1%) and flowered earlier (herbivore‐damaged = 83.9 ± 0.4 days, undamaged = 85.0 ± 0.4 days, LSMeans ± SE) than offspring of undamaged parent plants. Offspring of herbivore‐damaged plants also produced more flowers per plant than offspring of undamaged plants, although this effect was not statistically significant (*P* = 0.090; Table 5).

**Table 4:** Log‐likelihood ratio test of independence for the effect of herbivore‐damage treatment, maternal breeding type, their interaction, and maternal plant family on the proportion of *Solanum carolinense* offspring that flowered in the greenhouse and field. *P* values < 0.05 are in boldface.

Plant trait	Source of variation	df	*G* ^2^	*P*
Offspring flowering in greenhouse	Damage	1	4.089	**0.043**
	Breeding	1	0.772	0.380
	Breeding × Damage	3	5.458	0.141
	Family	2	8.010	**0.018**
Offspring flowering in field	Damage	1	0.190	0.663
	Breeding	1	3.625	0.057
	Breeding × Damage	3	9.140	**0.028**
	Family	2	3.546	0.170

**Table 5:** Linear mixed‐effects ANOVA for the effect of herbivore‐damage treatment, maternal breeding type, and their interaction on the average number of flowers and days to first flower of *Solanum carolinense* offspring in the greenhouse. *P* values < 0.05 are in boldface.

Plant trait	Source of variation	df	SS	*F*	*P*
Number of flowers	Damage	1	0.52782	2.89353	0.090
	Breeding	1	0.00003	0.00016	0.990
	Breeding × Damage	1	0.07627	0.41813	0.518
	Error	136	24.808		
Days to first flower	Damage	1	43.286	4.0940	**0.045**
	Breeding	1	1.006	0.0952	0.758
	Breeding × Damage	1	1.686	0.1595	0.690
	Error	136	1437.52		

There were no differences in the proportion of plants that flowered (Table 4), time to first flower (Table 5), or average number of flowers produced per plant (Table 5) between offspring of inbred and outbred maternal plants. There was also no effect of maternal plant family on average time to first flower [*χ* ^2^(1) = 0, *P* = 1] or number of flowers produced per plant [*χ* ^2^(1) = 0.364, *P* = 0.546] in the greenhouse. However, maternal plant family had a significant effect on the proportion of offspring that produced flowers (Table 4).

**Figure 3:**
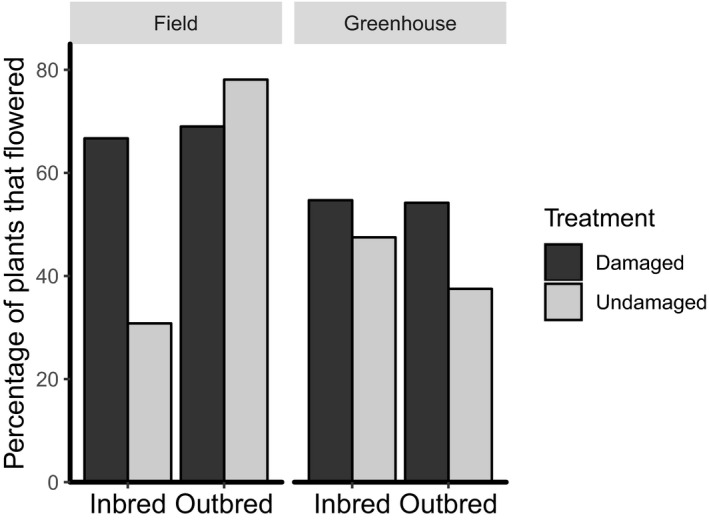
Proportion of *Solanum carolinense* offspring that flowered in the field and greenhouse.

There was no significant breeding by damage interaction effect on the proportion of plants that flowered (Table 4), average time to first flower (Table 5), or average number of flowers produced per plant (Table 5) in the greenhouse. A significantly greater proportion of offspring from herbivore‐damaged outbred plants flowered in the greenhouse compared to offspring of undamaged outbred plants (*G* ^2^ = 4.05, df = 1, *P* = 0.044; Fig. 3). However, there was no effect of previous generation herbivory on the proportion of plants that flowered from offspring of inbred maternal plants (*G* ^2^ = 0.64, df = 1, *P* = 0.424; Fig. 3).

### Maternal plant inbreeding negatively affects offspring flower production in the field

All plants in the field experienced high levels of herbivory, as solanaceous crop species (e.g., tomato, potato, and eggplant) were grown in nearby plots. Herbivory in the previous generation did not affect the proportion of plants that flowered (herbivore‐damaged = 68.2%, undamaged = 64.4%; Table 4) or the average number of flowers produced by offspring (Table 6) in the field. However, offspring of outbred maternal plants flowered at a higher proportion (outbred = 72.2%, inbred = 55%; Table 4) and produced significantly more flowers (Table 6) than offspring of inbred maternal plants. There was also a significant breeding by damage interaction effect on the proportion of plants that flowered (Table 4) and the average number of flowers produced per plant in the field (Table 6). Moreover, a significantly lower proportion of offspring from undamaged inbred plants produced flowers compared to all other treatments (Fig. 3). Maternal plant family did not have a significant effect on the proportion of plants that flowered (Table 4) or the average number of flowers produced per plant in the field (Appendix S1).

**Table 6:** Linear mixed‐effects ANOVA for the effect of herbivore‐damage treatment, maternal breeding type, and their interaction on the average number of flowers, average number of fruit, average number of seeds per fruit, and total number of seeds per plant of *Solanum carolinense* offspring in the field. *P* values < 0.05 are in boldface.

Plant trait	Source of variation	df	SS	*F*	*P*
Number of flowers	Damage	1	0.286	0.546	0.461
	Breeding	1	5.408	10.344	**0.002**
	Breeding × Damage	1	2.387	4.565	**0.035**
	Error	123	64.3		
Number of fruit	Damage	1	0.305	0.923	0.339
	Breeding	1	2.475	7.499	**0.007**
	Breeding × Damage	1	0.497	1.508	0.222
	Error	123	40.591		
Number of seeds per fruit	Damage	1	1518.89	3.189	0.080
	Breeding	1	966.43	2.029	0.160
	Breeding × Damage	1	388.88	0.816	0.371
	Error	50	23816.5		
Total number of seeds	Damage	1	127.8	0.717	0.401
	Breeding	1	646.92	3.630	0.062
	Breeding × Damage	1	57.86	0.325	0.571
	Error	50	8911.5		

### Herbivory in the previous generation has positive effects on offspring seed set in the field, while maternal plant inbreeding negatively affects offspring fruit production

**Figure 4:**
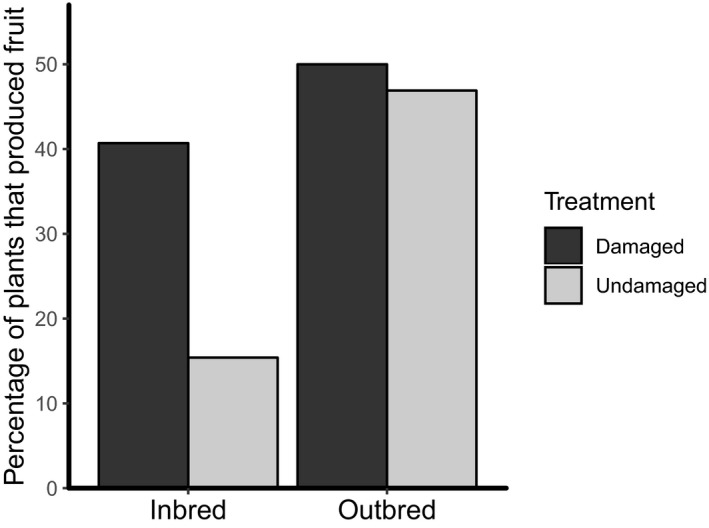
Proportion of *Solanum carolinense* offspring that produced fruit in the field.

Herbivory in the previous generation did not affect the total proportion of offspring that produced fruit in the field (herbivore‐damaged = 47.1%, undamaged = 37.8%; Table 7) or the average number of fruit produced per plant (Table 6). However, offspring of herbivore‐damaged plants produced more seeds per fruit than offspring of undamaged plants; however, this effect was not statistically significant (*P* = 0.080; Table 6). Offspring of outbred maternal plants produced significantly more fruit in the field than offspring of inbred maternal plants (Table 6). Furthermore, a greater proportion of offspring of outbred maternal plants produced fruit compared to offspring of inbred maternal plants, although this effect was not statistically significant (*P* = 0.079; outbred = 48.9%, inbred = 32.5%; Table 7). There was no difference in the average number of seeds produced per fruit between offspring of outbred and inbred maternal plants (Table 6). There was no significant breeding by damage interaction effect on the proportion of offspring that produced fruit in the field (Table 7); however, offspring of undamaged inbred plants produced fruit at a lower proportion than offspring of all other treatments (Fig. 4). There was also no breeding by damage interaction effect on the average number of fruit or seeds produced in the field (Table 6). No measure of reproductive output in the field was significantly affected by maternal plant family (Tables 4, 7; Appendix S1).

**Table 7:** Log‐likelihood ratio test of independence for the effects of herbivore‐damage treatment, maternal breeding type, their interaction, and maternal plant family on the proportion of *Solanum carolinense* offspring that produced fruit in the field. *P* values < 0.05 are in boldface.

Source of variation	df	*G* ^2^	*P*
Damage	1	1.0364	0.309
Breeding	1	3.0755	0.079
Breeding × Damage	3	5.9416	0.115
Family	2	0.35468	0.838

Herbivory in the previous generation did not affect the total number of seeds produced per plant in the field (Table 6). Offspring of outbred maternal plants produced more seeds per plant than offspring of inbred maternal plants; however, this effect was not statistically significant (*P* = 0.062; outbred = 895 ± 241, inbred = 404 ± 364, LSMeans ± SE; Table 6). There was no significant breeding by damage interaction effect on the total number of seeds produced per plant (Table 6). Maternal plant family had a slight effect on total number of seeds produced per plant, however it was not statistically significant (Appendix S1). For an overview of all our findings from this study, refer to Table 8.

**Table 8:** Summary of the statistical findings for each experiment performed in this study. X = offspring of outbred maternal plants; S = offspring of inbred maternal plants; DAM = parent plants subjected to repeated damage by *Manduca sexta* larvae; UD = parent plants were not damaged by *M. sexta* larvae (undamaged controls). *0.05 < *P* < 0.10; **0.01 < *P* < 0.05; *** *P* < 0.01; – *P* > 0.1. Superscript a (first column) indicates that separate statistical tests were run for the X and S plants to assess the effects of herbivore damage within each breeding type. The contents of each cell in the Mean emergence time and Time to first flower columns indicate which treatment emerged or flowered significantly earlier. The contents of the cells of all other columns indicate which treatment was significantly greater.

Source of variation	Greenhouse experiment							Field experiment					
	No. of seeds produced by maternal parents	Seed mass (mg)	Proportion of seedlings that emerged	Mean emergence time	Proportion of offspring that flowered	Time to first flower	Average no. of flowers	Proportion of offspring that flowered	Average no. of flowers	Proportion of offspring that produced fruit	Average no. of fruit	Average no. of seeds per fruit	Total no. of seeds per plant
Damage	–	–	*** DAM	*** DAM	** DAM	** DAM	* DAM	–	–	–	–	* DAM	–
Breeding	*** X	–	*** X	*** X	–	–	–	* X	*** X	* X	** X	–	* X
Breeding × Damage	–	–	***	***	–	–	–	**	**	–	–	–	–
Family	–	–	***	–	**	–	–	–	–	–	–	*	*
X‐DAM vs. X‐UD^a^	–	–	*** X‐DAM	–	** X‐DAM	–	–	–	–	–	–	–	–
S‐DAM vs. S‐UD^a^	–	–	*** S‐DAM	** S‐DAM	–	–	–	** S‐DAM	–	* S‐DAM	–	–	–

## DISCUSSION

Our results indicate that both herbivory and maternal plant inbreeding have a transgenerational impact on traits associated with offspring fitness in *Solanum carolinense*. We found that offspring of herbivore‐damaged plants had greater seedling emergence, emerged more rapidly, flowered earlier, and produced more flowers and seeds than offspring of undamaged plants (Table 8). In addition, we found that maternal plant inbreeding adversely impacts herbivore‐induced transgenerational effects even though the offspring are outbred. We also found that the fitness consequences of these transgenerational effects depend on the environmental conditions experienced by offspring, including the presence of herbivores (Table 8).

### Herbivory and maternal plant inbreeding affect seed production and seedling emergence

We found that *Manduca sexta* herbivory of parent plants positively affected seedling emergence in *S. carolinense,* with seedlings from herbivore‐damaged plants emerging at a higher proportion and earlier than seedlings from undamaged plants (Table 8). Previous studies have found both positive and negative effects of herbivory on seed set, seed mass, and seedling viability (Crawley and Nachapong, 1985; Weiner et al., 1997; Agrawal, 1998; Moreira et al., 2015). Additionally, there is some evidence that herbivore damage to a parent plant can result in increased seed germination and seedling emergence (Karban and Lowenberg, 1992; Moreira et al., 2015; Alba et al., 2016). We also found that inbred maternal plants produced fewer total seeds than outbred plants, and that maternal plant inbreeding negatively affected the timing and total proportion of seedling emergence. These results are consistent with numerous studies showing that inbred plants generally produce fewer, often smaller, and less viable seeds than outbred plants (Husband and Schemske, 1996; Baskin and Baskin, 2015).

While many studies have shown that maternal environmental conditions mediate offspring provisioning and impact offspring performance (see review by Roach and Wulff, 1987; Agrawal, 2001), others have shown that seed and seedling traits can be independent of effects mediated by the maternal environment of a plant (Agrawal, 2002; Alba et al., 2016). Quantitative differences in seed provisioning are unlikely to explain differences in seedling emergence in our study, since there were no differences in seed mass between herbivore‐damaged and undamaged plants or between outbred and inbred plants (Table 8). There could, however, still be differences in the quality of the resources provided by the parental plants. For example, maternal herbivory could result in the accumulation of phytohormones in plant seeds that affect seed dormancy. Singh et al. (2017) showed that seeds from herbivore‐damaged *Arabidopsis* plants had a higher germination rate than seeds from undamaged plants and that this difference was the result of jasmonic acid (JA) accumulation in the seeds. JA has also been associated with seed dormancy and germination in a variety of other plant species; however, its roles remain poorly understood (Linkies and Leubner‐Metzger, 2012; Wasternack et al., 2013). While we did not explicitly measure JA concentrations in *S. carolinense* seeds, it is well known that herbivory by chewing insects, such as *M. sexta*, upregulates JA production in a wide variety of species (Howe and Jander, 2008), including *S. carolinense* (Campbell et al., 2014). Furthermore, seed dormancy and germination are also regulated by maternally derived mRNAs and proteins (see review by Donohue, 2009) that are known to be impacted by herbivory (Hui et al., 2003; Giri et al., 2006).

### Maternal inbreeding effects are more severe under harsher environmental conditions

The effects of plant inbreeding on within‐generation fitness have been extensively studied, and research has unambiguously shown that inbreeding negatively affects plant growth, development, and reproduction (Charlesworth and Charlesworth, 1987; Husband and Schemske, 1996). All seeds produced in this study, however, were from cross‐pollinations; thus, all offspring should have an inbreeding coefficient of 0 (ƒ = 0), and any phenotypic differences should be independent of the effects of homozygosity on inbreeding depression in the offspring generation (Hartl and Clark, 2007).

We found a negative effect of maternal plant inbreeding on seedling emergence. Furthermore, in the field, offspring of inbred maternal plants had a significantly lower reproductive output than offspring of outbred maternal plants (including lower flower and fruit production) and inbreeding had a nearly significant negative effect on total seed production per plant (Table 8). These results indicate that the effects of inbreeding on plant fitness (i.e., inbreeding depression) can extend to outbred offspring and that maternal inbreeding can have negative consequences on offspring reproduction. This finding is surprising because maternal environmental effects tend to be pronounced during early stages of offspring development (i.e., germination and seedling establishment) but diminish during later stages of growth (e.g., Roach and Wulff, 1987). Consequently, the mechanism underlying the transgenerational impact of maternal inbreeding on offspring reproduction is likely to be due to something more than small differences in nutrient or energy storage in the seeds.

We also found that the negative effects of maternal plant inbreeding on reproductive traits were stronger in the field than in the greenhouse. Previous studies have shown that inbreeding depression can have more severe consequences for plant fitness under harsher environmental conditions (Armbruster and Reed, 2005; Hayes et al., 2005; Fox and Reed, 2010), but these studies did not examine next‐generation effects on outbred offspring. The current results indicate that studies conducted in controlled greenhouse settings may underestimate the level of transgenerational inbreeding depression that would be observed under more natural environmental conditions. Such differences in phenotypic responses to different environments demonstrates the need to conduct both field and greenhouse experiments when trying to understand the transgenerational effects of herbivory and/or inbreeding on plant fitness components.

### Transgenerational effects of herbivory are environmentally dependent

Our results show that the effects of previous‐generation herbivory on reproductive traits in *S. carolinense* also depend on the environment experienced by offspring. In our greenhouse studies, we found that herbivory not only positively affected seedling emergence, but that offspring of herbivore‐damaged plants also flowered at a higher proportion, flowered earlier, and produced slightly more flowers than offspring of undamaged, control plants (Table 8). The effects of previous generation herbivory on offspring reproductive output were less apparent when offspring were grown in the field, where herbivores inflicted high levels of damage. While we did not see any differences in flower or fruit production between offspring of herbivore‐damaged and undamaged parents in the field, we did observe transgenerational effects of herbivory on the number of seeds produced per fruit (Table 8).

Few previous studies have examined the transgenerational effects of herbivory on offspring growth and reproductive traits (Agrawal, 2001, 2002; Steets and Ashman, 2010; Holeski et al., 2013; González‐Megías, 2016). Moreover, the effects reported are inconsistent; some studies demonstrated positive maternal effects of herbivory on offspring growth (Agrawal, 2002; Steets and Ashman, 2010), others showed negative effects (González‐Megías, 2016), and some showed no effect at all (Holeski et al., 2013). For example, Steets and Ashman (2010) found that *Impatiens capensis* plants that experienced natural levels of herbivory produced significantly larger offspring, which produced more flowers, compared to offspring of plants protected from herbivores, while González‐Megías (2016) found that floral and root herbivory on maternal plants reduced seedling emergence and establishment of *Moricandia moricandioides* in the field. The findings from our study, which examined offspring performance in both the greenhouse and the field and found that the transgenerational impact of herbivory was dampened under field conditions, suggest that the inconsistencies reported in the literature may be due, at least in part, to differences in the environments in which the offspring were assessed.

### Maternal effects and epigenetic modifications may contribute to transgenerational responses of plants

Mechanistically, transgenerational effects of plant herbivory and inbreeding could be due to the combination of maternal environmental effects (i.e., seed provisioning) and epigenetic modifications to the offspring phenotype. While this study did not reveal an association between seed mass and seedling vigor, other effects of the maternal environment on seed traits, such as maternal resource provisioning to the seed coat and seed endosperm, which are known to be important factors in seed dormancy and germination, could be influenced by herbivory and inbreeding (Roach and Wulff, 1987; Donohue and Schmitt, 1998). Additionally, the transmission of maternally derived mRNA transcripts, proteins, and hormones could affect offspring life history traits and phenotypes (Frey et al., 2004; Finch‐Savage and Leubner‐Metzger, 2006; Donohue, 2009).

Transgenerational effects of the parental environment on offspring phenotype might also be caused by stress‐induced epigenetic modifications of the offspring genome (Jablonka and Raz, 2009; Hauser et al., 2011; Herman and Sultan, 2011; Holeski et al., 2012). Epigenetic modifications, primarily DNA methylation and histone modifications, alter the patterns and magnitude of gene expression and are known to influence plant growth and reproduction (Pikaard and Scheid, 2014; Campos‐Rivero et al., 2017), plant response to stress (Chinnusamy and Zhu, 2009; Thiebaut et al., 2019), and the transgenerational induction of plant defenses (Holeski et al., 2012). It is possible that epigenetic modifications of the plant genome contribute to the independent transgenerational impact of herbivory that we observed. Recent evidence also suggests that epigenetic modifications of the plant genome are involved in plant inbreeding depression (Vergeer et al., 2012), which could potentially contribute to the observed effects of inbreeding on transgenerational responses to herbivory. Vergeer et al. (2012) found that *Scabiosa columbaria* inbred plants had higher levels of DNA methylation compared to outbred plants and demonstrated that inbreeding depression disappears after treatment with the demethylation agent. They found that when 5‐azacytidine treatment restored DNA methylation levels of inbred plants to that of outbred plants, there was an increase in the photosynthetic efficiency, number of leaves, and biomass of inbred plants. Should DNA methylation (1) mediate the negative effects of inbreeding (Vergeer et al., 2012) and (2) have the potential to persist across generations, as is known to occur for biotic and abiotic stresses (Chinnusamy and Zhu, 2009; Herman and Sultan, 2011; Holeski et al., 2012), then epigenetic modifications influenced by inbreeding may explain our finding that the effects of inbreeding depression can persist into the next generation even among outbred progeny. Unfortunately, the design of the current study does not allow us to separate maternal from paternal epigenetic modifications based on herbivore damage, as pollen from herbivore‐damaged plants was used to produce the seeds on herbivore‐damaged maternal plants, while pollen from undamaged plants sired the seeds on undamaged maternal plants.

## CONCLUSIONS

This study documents transgenerational effects of herbivory and plant inbreeding on components of *S. carolinense* fitness (e.g., seedling emergence, flower production, fruit production, and seed production) in the greenhouse and field. We found positive transgenerational effects of herbivory and negative effects of inbreeding on seedling emergence and offspring reproductive output (even though all offspring plants in our study were outbred). Furthermore, our results reveal that these transgenerational effects of herbivory and maternal plant inbreeding depend on the environment experienced by offspring. Our findings also suggest that inbreeding can compromise transgenerational responses to herbivory. While this research contributes to the growing literature on the transgenerational impacts of herbivory on offspring fitness, future research should implement sophisticated experimental designs to further elucidate parental contributions to the epigenome of their offspring.

## AUTHOR CONTRIBUTIONS

C.T.N., A.G.S., M.C.M., and C.M.D. conceived and designed the experiments. C.T.N., W.S.W., and S.J.B. carried out the experiments. C.T.N. analyzed the data. C.T.N., A.G.S., M.C.M., and C.M.D. wrote and revised the manuscript.

## DATA AVAILABILITY

The raw data for each figure and table in this manuscript are available at Pennsylvania State University's ScholarSphere data repository (https://doi.org/10.26207/bps0-at80).

## Supporting information


**APPENDIX S1.** Likelihood ratio tests performance models for linear mixed‐effects model ANOVAs with and without the random family effect on the reproductive output of *Solanum carolinense* offspring in the field. *P‐*values indicated whether maternal plant family was a signfiicant random effect in the model.
