# Supplementary material for: Transgenerational impacts of herbivory and inbreeding on reproductive output in Solanum carolinense
Source: Am J Bot. 2020 Jan 15;107(2):286–97. doi: 10.1002/ajb2.1402 (PMC7064912; doi:10.1002/ajb2.1402)
Supplement: Supplementary file 1 — APPENDIX S1. Likelihood ratio tests performance models for linear mixed‐effects model ANOVAs with and without the random family effect on the reproductive output of Solanum carolinense offspring in the field. P‐values indicated whether maternal plant family was a signfiicant random effect in the model. [file AJB2-107-286-s001.docx]

Appendix S1. Likelihood ratio tests performance models for linear mixed-effects model ANOVAs with and without the random family effect on the reproductive output of *Solanum carolinense* offspring in the field. *P* values indicated if maternal plant family was a signfiicant random effect in the model.

| Trait | df | *χ*^2^ | *P* |
| --- | --- | --- | --- |
| Number of flowers | 1 | 0.0527 | 0.818 |
| Number of fruit | 1 | 0.000 | 1 |
| Number of seeds per fruit | 1 | 0.962 | 0.327 |
| Total number of seeds per plant | 1 | 3.297 | 0.069 |
